# Impact of Obesity and Lysosomal Dysfunction on Chemoresistance in Ovarian Cancer

**DOI:** 10.3390/biomedicines12030604

**Published:** 2024-03-07

**Authors:** Boyun Kim, Jewon Jung

**Affiliations:** Department of SmartBio, College of Life and Health Science, Kyungsung University, Busan 48434, Republic of Korea; boyunism@gmail.com

**Keywords:** obesity, ovarian cancer, lysosome, chemoresistance, adipocyte, tumor microenvironment, lipid metabolism

## Abstract

Obesity is recognized as a significant risk factor for ovarian cancer, with accumulating evidence highlighting its impact on disease progression and chemoresistance. This review synthesizes current research elucidating the link between obesity-induced lysosomal dysfunction and ovarian cancer chemoresistance. Epidemiological studies consistently demonstrate a positive correlation between body mass index (BMI) and ovarian cancer risk, attributed in part to the predilection of epithelial ovarian cancer cells for adipose tissue, particularly the omentum. Adipokines released from the omentum contribute to cancer-associated characteristics, including energy supply to cancer cells. Moreover, obesity-induced alterations in lysosomal function have been implicated in systemic inflammation and lipid metabolism dysregulation, further exacerbating cancer progression. Lysosomes play a crucial role in drug resistance, as evidenced by studies demonstrating their involvement in mediating resistance to chemotherapy in ovarian cancer cells. Recent findings suggest that pharmacological inhibition of lysosomal calcium channels sensitizes drug-resistant ovarian cancer cells to cisplatin treatment, highlighting the therapeutic potential of targeting lysosomal dysfunction in obesity-related chemoresistance. This review underscores the importance of understanding the multifaceted roles of lysosomes in obesity-related drug resistance and their implications for the development of targeted therapeutic interventions in ovarian cancer management.

## 1. Introduction

Ovarian cancer, ranking as the fifth leading cause of cancer-related deaths in women, exceeds all other cancers affecting the female reproductive system in terms of mortality. Accounting for 5% of female cancer-related deaths within gynecological cancers, ovarian cancer is characterized by high lethality [1]. Challenges in early detection and the development of chemoresistance significantly contribute to this outcome. The standard treatment for ovarian cancer involves a combination of platinum-based drugs and taxanes. While the initial response to this standard therapy is positive in the majority of ovarian cancer patients, recurrence occurs in up to 80% of cases, primarily due to the development of platinum resistance [2]. The presence of chemoresistance to the standard treatment poses a crucial obstacle in the effective management of ovarian cancer, leading to a decline in the 5-year survival rate [3,4]. Various factors, including tumor heterogeneity, microenvironmental effects, and disruptions in drug access to the target compartment, contribute to the development of resistance to chemotherapeutics [5,6].

Several years ago, the American Institute for Cancer Research and World Cancer Research Fund reached the consensus that the accumulation of body fats poses a potential risk factor for ovarian cancer. Numerous systemic studies and meta-analyses have consistently revealed a positive correlation between body mass index (BMI) and ovarian cancer risk [7,8,9,10,11]. From a physiological perspective, epithelial ovarian cancer cells predominantly disseminate within the abdominal cavity, displaying a predilection for adipose tissue, particularly the omentum. Omental metastasis is detected in approximately 80% of patients with serous ovarian carcinoma [12]. The inclination of this preference for adipose tissue is believed to be rooted in the release of adipokines from the omentum, which, in turn, promotes cancer-associated characteristics by supplying energy from adipocytes to cancer cells [13]. In the context of chemoresistance, lipids or their regulatory factors derived from adipose tissues have been reported to affect the survival of ovarian cancer cells, as well as influencing proliferation or metastasis [14,15,16].

Numerous studies have suggested the link between obesity and cellular processes passing through lysosomes in a variety of aspects. Defective functions of lysosomes in adipocytes cause impairment of autophagic flux through an imbalance in lysosomal calcium, consequently activating a systemic inflammation in obese mice [17]. Additionally, obesity not only activates lysosomal functions in adipocytes but also triggers lysosomal biogenesis in macrophages residing in adipose tissue. This upregulation in lysosomal activity in macrophages is closely associated with lipid catabolism, contributing significantly to the development of obesity-induced inflammation and lipid trafficking independent of the inflammatory response [18]. In addition to their role in obesity-related inflammation, lysosomes play a crucial role in drug resistance, particularly in various cancers, including ovarian cancer. Our previous study provided insights into the involvement of lysosomes in mediating drug resistance, specifically in ovarian cancer cells [19]. Notably, our unpublished data demonstrated that pharmacological inhibition of lysosomal calcium channels sensitizes drug-resistant ovarian cancer cells to cisplatin treatment. The intricate interplay between lysosomes and cellular processes underscores their significance in various physiological and pathological conditions. This review highlights the impact of lysosomal dysfunction induced by obesity on ovarian cancer chemoresistance. Furthermore, understanding of the association between lysosomes and obesity, as well as their involvement in obesity-related drug resistance, can provide valuable insights into potential therapeutic interventions for ovarian cancer.

## 2. Obesity and Ovarian Cancer

Epidemiological findings suggest that obesity has a detrimental effect on the development of ovarian cancer [7,20,21]. Obesity is defined as a pathophysiological condition characterized by the accumulation of excessive body fat and a chronic low-grade inflammatory state to the extent that it may have adverse effects on health [22]. Specifically, the association between obesity and ovarian cancer risk appears to be dependent on certain histologic types of cancer, including low-grade serous and invasive mucinous tumors [20]. The excessive accumulation of adipose tissue can alter the metabolism of chemotherapeutic agents, and subsequently reduces chemotherapy efficacy [23,24]. The distribution and clearance of drugs may be different in obese individuals, potentially leading to suboptimal drug concentrations at the tumor site [25]. The relationship between obesity and chemoresistance in ovarian cancer is an area of ongoing research, and, while the exact mechanisms are not fully understood, several factors may contribute to the association between obesity and reduced responsiveness to chemotherapy in ovarian cancer patients.

### 2.1. Adiposity and Ovarian Cancer Cells

**Figure 1:**
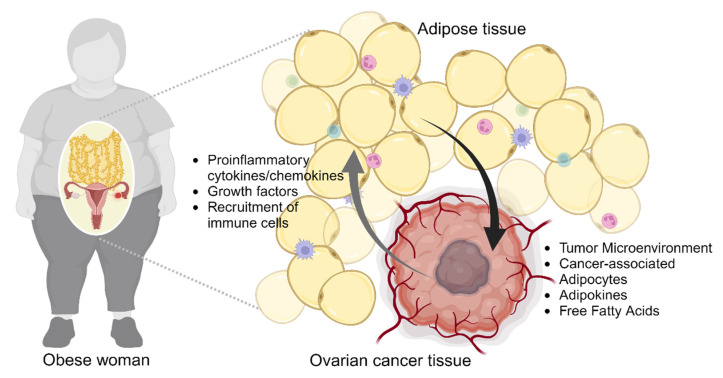
Bidirectional interaction between ovarian cancer tissue and adipose tissue in obese women. The promoting effects of adipose tissue on ovarian cancer in obese women are notable within the tumor microenvironment, where cancer-associated adipocytes release adipokines and free fatty acids, creating a pro-inflammatory milieu that supports tumor progression and chemoresistance. Tumor tissue stimulates differentiation of adipocytes into cancer-associated adipocytes by secreting pro-inflammatory and growth factors.

Adiposity arises from an imbalance between an overabundance of calorie consumption and insufficient energy expenditure, leading to a persistent surplus of energy [26]. This imbalance triggers immune cell infiltration and disrupts the controlled secretion of various adipokines, ultimately causing dysfunctional adipose tissue [27]. Metabolic stress stimulated by dysfunctional adipose tissue, in accordance with immune cells, has the potential to support tumor growth and metastasis [27,28]. Furthermore, adiposity has recently emerged as a plausible risk factor associated with diminished chemotherapy effectiveness, contributing to the development of chemoresistance [24]. In ovarian cancer, the accumulation of adipose tissue induces hormonal alterations and ovulatory dysfunction, leading to excessive synthesis of ovarian and extra-ovarian estrogen. This may subsequently become a significant risk factor in ovarian cancer development [29,30]. While metastatic ovarian cancer cells from the primary tumor potentially disseminate throughout the peritoneal cavity, the most common arriving site is the omentum, which consists of a large number of adipocytes [12]. This is supported by the observation that 80% of serous ovarian cancer patients exhibit omental metastasis [13,31]. The omentum, an adipocyte-rich tissue, likely aids ovarian cancer cells in adapting to a new environment distinct from the primary site. Various adipokines, including IL-6, IL-8, and fatty acids, secreted from the omental adipocytes enhance the adaptation of ovarian cancer cells by promoting proliferation and metastasis (Figure 1) [13,32].

Abdominal or central obesity has been implicated in various health issues including ovarian cancer. Central obesity can be assessed using a waist-to-hip ratio (WHR) estimating anatomic distribution of fats. Some studies have explored the relationship between central obesity and ovarian cancer risk [21,29,33]. Delort et al. indicate that there is an association between BMI and ovarian cancer risk that varies depending on menopausal status [29]. Specifically, higher BMI appears to have a stronger association with ovarian cancer risk in premenopausal women compared to postmenopausal women [29]. Along with the results, they discovered a significant link between WHR and ovarian cancer risk which remained consistent regardless of menopausal status [29]. Hoyo et al. also reported that WHR, weight, and BMI have a link with increased ovarian cancer risk across African American women and White women [33]. While, interestingly, the association between WHR and ovarian cancer risk was found in African American women, no risk appeared in White women [33]. Based on these studies, WHR estimating fat distribution, as well as BMI, should be considered as a factor to determine ovarian cancer risk. However, there is little direct evidence of the association of ovarian cancer chemoresistance with WHR.

Obesity exerts an influence on cancer development; however, there is an instance where an augmentation in peritumoral adipocytes impacts cancer progression irrespective of BMI or WHR. Zhang et al. reported that peri-renal adiposity, independent of body mass index (BMI) or body fat distribution, negatively affected rates of progression-free survival in ovarian cancer [34]. Additionally, a recent study has reported that bone marrow adipocytes provide favorable conditions for invasion in bone metastasis cases and facilitate chemoresistance through supporting cancer-associated fibroblasts [35]. Transcriptomic analysis of bone marrow metastasized cancer cells compared to primary ones in the same patients revealed upregulation of drug resistance and immune evasion-related gene sets affected by bone marrow adipocytes [35]. In several ovarian cancer cell lines, when exposed to adipocyte-conditioned medium, they exhibited increased survival against cisplatin-induced cell death via activation of the AKT pathway [15]. Lipidomic analysis of adipocyte-conditioned medium revealed that arachidonic acid secreted from adipocytes contributes to the heightened chemoresistance observed in ovarian cancer cell lines [15]. Kim et al. reported that anatomical differences of adipose tissue demonstrated genetical distinctions in their stromal cells derived from subcutaneous and visceral fats, impacting energy and lipid metabolism [36]. These unique characteristics of subcutaneous and visceral adipose stromal cells (ASCs) also exert different effects on neighboring ovarian cancer cells. The conditioned medium (CM) from visceral ASCs stimulates increased migration of ovarian cancer cells by activating JAK2-STAT3 through the secretion of IL-6 in a paracrine signaling pathway, in contrast to the results observed with CM from subcutaneous ASCs [37]. Likewise, human omental-derived ASCs enhance the survival and resistance response of ovarian cancer cells to chemotherapy, such as paclitaxel or carboplatin treatment [38].

### 2.2. Obesity-Induced Remodeling of Tumor Microenvironment

Cancer cells adeptly manipulate their microenvironment to withstand adverse conditions such as hypoxia and nutrient deprivation. Extensive research has scrutinized the intricate interplay between cancerous tissue and its microenvironment, comprising diverse cellular components like stromal cells, inflammatory cells, and fibroblasts [39]. Notably, adipocytes, traditionally perceived as mere energy reservoirs, have emerged as crucial endocrine entities within the tumor milieu [40]. Despite their emerging significance in cancer research, adipocytes’ involvement in the tumor microenvironment remains largely underexplored. Adipocytes intricately modulate cancer progression, metastasis, and drug responses, playing dynamic roles in tumor biology [41]. Peritumoral or intratumoral adipocytes, referred to as cancer-associated adipocytes, exhibit distinctive phenotypic traits and molecular features, including altered marker expression and reduced lipid content, thus promoting metabolic reprogramming of tumor cells [42,43]. These altered adipocytes secrete fatty acids, providing essential energy substrates that fuel metabolic shifts in cancer cells [41,44]. Free fatty acids facilitate restructuring of the tumor microenvironment, thereby contributing to cancer progression and metastasis [44].

Additionally, obesity exerts profound effects on the composition and functionality of the tumor microenvironment [45]. Perturbations in this milieu may foster a conductive niche for cancer cell survival and compromise chemotherapy efficacy by releasing metabolites and growth factors from intratumoral or peritumoral adipocytes. High-fat-diet-induced obesity impairs the function of infiltrating CD8^+^ T cells within the tumor microenvironment, consequently accelerating tumor progression in murine models. Notably, single-cell analyses underscored the global metabolic rewiring observed in tumors under high–fat-diet conditions [46]. This investigation elucidated distinct metabolic states across diverse cell populations within the tumor microenvironment, illustrating their mutual reliance. In ovarian cancer, fatty acid binding protein 4 (FABP4) secreted by co-cultured adipocytes critically modulates lipid responses in ovarian cancer cells. Consequently, pharmacological inhibition of FABP4 using the small molecule BMS309403 mitigates tumor metastasis and enhances the sensitivity of cancer cells to carboplatin [14]. Moreover, obesity induced by a high-fat diet alters the M1/M2 macrophage ratio and promotes tumor fibrosis through the upregulation of fibroblast growth factor 21 (FGF21), thereby diminishing the efficacy of standard chemotherapy treatments such as paclitaxel and carboplatin in murine models [47]. The overexpression of FGF21 is observed in cisplatin-resistant ovarian cancer cell line A2780-cp compared to cisplatin-sensitive cell line A2780-s [48]. Similarly, elevated EGF21 levels and tumor fibrosis are detected in human ovarian tumor tissues from patients with a BMI > 30 [47]. It is crucial to highlight that the relationship between obesity and chemoresistance is complex, and individual responses may vary. The impact of obesity on cancer treatment outcomes is likely influenced by a combination of factors, including the specific characteristics of the tumor, the type of chemotherapy used, and the overall health status of the patient.

### 2.3. Lipid Metabolism in Ovarian Cancer

In patients with obesity, it is likely that the combination of enhanced mitogenic and growth factor signaling in response to the altered hormonal milieu and the increased availability of carbon-rich nutrients, such as lipids and glucose, supports biomass production and proliferation, thereby accelerating disease progression and treatment resistance. In the case of cancer, the cells undergo metabolic reprogramming to support their rapid proliferation, resistance to cell death, and progression [49]. Lipids constitute a diverse class of biological molecules, encompassing fatty acids, glycerides, non-glyceride lipids, and complex lipids. In cancer, lipid metabolism assumes a significant role by offering metabolic fuels for mitochondrial oxidation, substrates for phospholipid synthesis, and other signaling molecules [50]. Altered lipid metabolism, a common feature in many human cancers, contributes to the increased synthesis of lipids and upregulation of associated enzymes and signaling pathways. This process provides essential building blocks for maintaining cellular membranes, such as glycerophospholipids, and facilitates the production of signaling molecules that play a role in promoting oncogenic signals, such as diacylglycerol [51,52].

Epidemiological evidence suggests that people with the highest BMI have a higher risk of dying from cancer than those with normal BMI [22]. Excessive adiposity resulting from obesity added to the enhanced mitogenic and growth factor signaling in cancers is likely to accelerate cancer progression and chemoresistance, causing a low survival rate in cancer patients [52,53]. In the case of ovarian cancer, stage-specific effects of obesity on survival rate were observed in several studies [11,21,22,53]. The biological features of adipose tissues dominantly composed of adipocytes are dramatically modified by obesity. These alterations additionally affect lipid metabolism of adipocytes, resulting in effects such as excessive release of fatty acids and secretion of adipokines. Free fatty acids utilized as a key energy source are excessively released in response to metabolic abnormalities such as obesity, thereby accelerating disease progression and chemoresistance [54]. In the context of ovarian cancer chemoresistance and obesity, inhibition of a lipid chaperon protein sensitizes ovarian cancer cells to carboplatin treatment both in vitro and in vivo [14]. A comprehensive lipidomic analysis indicated that lipids secreted from both subcutaneous and visceral adipocytes enhance cell survival of cisplatin-induced apoptosis through directly activating AKT in ovarian cancer cells [15]. Management of obesity and its associated metabolic effects may be important in improving treatment outcomes in ovarian cancer patients. However, more research is needed to better understand the underlying mechanisms and to develop targeted strategies to address chemoresistance in obese individuals with ovarian cancer.

Understanding the molecular mechanisms underlying the interplay between obesity and chemoresistance could pave the way for the development of novel therapeutic strategies tailored to obese cancer patients. Additionally, implementing lifestyle interventions targeting weight management and metabolic health may complement traditional cancer treatments and improve clinical outcomes in obese individuals undergoing chemotherapy [55]. Furthermore, ongoing research efforts focusing on precision medicine approaches aim to elucidate patient-specific factors that influence the response to chemotherapy in the context of obesity, thereby advancing personalized cancer care strategies. Ultimately, a multifaceted approach considering the complex interplay between obesity, tumor biology, and treatment modalities is essential for optimizing therapeutic outcomes and improving the prognosis of obese cancer patients.

## 3. Lysosomal Dysfunction in Obesity and Cancer

### 3.1. Lysosomal Dysfunction in Diseases

The lysosome, a single-membrane organelle characterized by its acidic environment, was initially identified in 1955 by Christian de Duve during investigations into insulin’s mechanism of action [56,57]. Housing more than 60 hydrolytic enzymes, including nucleases, glycosidases, phosphatases, sulfatases, lipases, and proteases, the lysosome operates within a luminal acidic milieu (pH 4.5–5.0) maintained by the vacuolar ATPase (V-ATPase) proton pump [58,59]. Traditionally acknowledged as the hub for waste disposal, lysosomes undertake the digestion of unwanted macromolecules, damaged and senescent organelles, microbes, and particles acquired through endocytosis, autophagy, and phagocytosis [60,61,62]. After degradation, specific products, such as free fatty acids, amino acids, monosaccharides, and nucleotides, are transported back to the cytosol via dedicated lysosomal membrane exporters for reuse in anabolic processes [63,64]. Additionally, lysosomes house more than 60 membrane proteins crucial for maintaining luminal homeostasis, particularly in ionic balance, membrane potential, molecular export, and lysosomal membrane trafficking (fusion and fission) [65]. The essential functions of lysosomes in material degradation, catabolite export, and trafficking are pivotal for cellular homeostasis, and disruptions often lead to lysosomal storage diseases (LSDs) [66].

Recent investigations challenge the traditional view of the lysosome as merely a degradative compartment, revealing its role as a multifunctional signaling hub that integrates cellular responses to nutrient status, growth factors, and hormones [67,68,69]. Notably, the lysosome employs a nutrient-sensing mechanism involving mammalian/mechanistic target of rapamycin complex 1 (mTORC1) and transcription factor EB (TFEB) to adapt to changes in the cellular environment [70,71,72]. mTORC1, sensitive to various nutrient and energy cues, phosphorylates numerous substrates related to cell growth, including TFEB, thereby regulating the balance between catabolic and anabolic metabolic pathways [73]. TFEB, capable of binding to the palindromic 10 bp nucleotide motif known as the coordinated lysosomal expression and regulation (CLEAR) element, activates the transcription of genes encoding lysosomal proteins and autophagy-related proteins [74]. Under nutrient-rich conditions, mTORC1 phosphorylates TFEB, sequestering it away from the nucleus. Conversely, during starvation, TFEB undergoes dephosphorylation due to reduced mTORC1 activity and the activation of calcineurin (CaN), a Ca^2+^ and calmodulin (CaM)-dependent protein phosphatase. This dephosphorylation allows TFEB to translocate to the nucleus, promoting the transcription of the CLEAR element and subsequently enhancing the autophagy–lysosome pathway, exocytosis, and phagocytosis [75,76,77].

### 3.2. Lysosomal Dysfunction in Diseases

Considering the integral roles of lysosomes in cellular metabolism, proliferation, differentiation, immunity, and programmed cell death, any alteration or dysfunction in lysosomal activity has the potential to disrupt the inherent homeostasis of cells and organisms, contributing to the onset or exacerbation of human diseases. Dating back to the 1960s, H. G. Hers elucidated the association between lysosomal α-glucosidase deficiency and Pompe disease, pioneering the concept of inborn lysosomal diseases, exemplified as lysosomal storage disorders (LSDs) [78]. LSDs constitute a rare group of metabolic disorders stemming from inherited mutations in genes encoding proteins crucial for lysosomal homeostasis, including lysosomal hydrolases or membrane proteins [79]. Beyond LSDs, numerous diseases such as cancer, obesity, diabetes, neurodegenerative diseases, and cardiovascular diseases have been shown to exhibit close correlations with lysosomal alterations and dysfunction [80,81,82,83,84,85,86]. This review delves into obesity and cancer, exploring their lysosomal changes and dysfunction to establish a foundation for the subsequent selection of targeted therapeutic strategies.

#### 3.2.1. Obesity

Lysosome dysfunction in obesity is linked to various cellular processes, contributing to adverse health effects. Luo et al. investigated the intricate relationship between CD36, lysosome function, and the development of obesity-related metabolic complications [17]. The research demonstrates a dual phenomenon in visceral adipose tissue pre-adipocytes obtained from obese individuals or mice on an obesogenic diet: an upregulation of CD36 expression and a reduction in acidified lysosome abundance. Intriguingly, CD36 global null mice on a high-fat diet exhibit restrained pre-adipocyte expansion, increased acidified lysosomes, reduced inflammatory markers, and improved glucose tolerance and insulin sensitivity. In cell culture studies, the researchers revealed that fatty acids induce CD36 upregulation in pre-adipocytes [17,87,88]. Furthermore, forced expression of CD36 triggers lysosomal pH abnormalities, inflammatory cytokine production, and impaired lipophagy. Experiments with exogenously expressed wild-type CD36 highlight its increased interaction with tyrosine kinase Fyn, elevated IP3R1 phosphorylation, co-localization of Ca^2+^ with lysosomes, and heightened cytokine production compared to its palmitoylation-deficient mutant [17]. Pharmacological inhibition of IP3R1 and Fyn attenuates this phenotype, implicating the involvement of CD36/Fyn/IP3R1 signaling in lysosomal Ca^2+^ content increase and pH impairment [17,89]. These findings provide compelling evidence supporting the notion that obesity induces acquired lysosome dysfunction, leading to inflammation and suggesting potential therapeutic targets in cardio-metabolic diseases. CD36 acts as a scavenger receptor facilitating the uptake of lipids, such as long-chain fatty acids and lipoproteins [90]. Transcriptionally upregulated in pre-adipocytes via PPARγ signaling, CD36 drives adipose tissue expansion [91]. The upregulation of CD36 in hepatocytes and other cell types in response to a high-fat diet is linked to pleiotropic signaling, negatively regulating autophagy through Fyn-induced phosphorylation of LKB1. CD36 deficiency induces lipophagy, protecting mice from high-fat-diet-induced obesity [91], mirroring findings presented by Luo et al. [17]. CD36 deficiency also correlates with increased nuclear translocation of TFEB [92], suggesting a role in stimulating lysosome function and the observed benefits. The study proposes that homeostatic lysosome function in adipocytes may link lipolysis to exosome release, influencing adipose tissue macrophages and upregulating the lysosome biogenesis program to maintain homeostasis and modulate inflammation [93]. Inborn errors of metabolism resulting in lysosome storage diseases demonstrate loss of adipose tissue and inflammation, paralleling the mechanistic link observed in mice with diet-induced obesity [17]. Conversely, stimulation of lysosome biogenesis and function in adipocytes, activating TFEB, attenuates diet-induced obesity and improves insulin sensitivity.

#### 3.2.2. Cancer

Cancer cells demonstrate a remarkable ability to manipulate lysosomal dynamics to fuel their growth and proliferation [94]. This intricate regulation involves changes in lysosomal quantity, location, and activity, driven by the overexpression of specific lysosomal proteins and lysosome-related proteins such as lysosome catalase, lysosomal glycosidase, and kinesins [95,96,97]. Classical oncogenes like *KRAS* and *MYC* further contribute to this transformation [95]. Notably, certain cancers, including pancreatic adenocarcinoma [81,98], renal cell carcinoma [98], melanoma [99], head and neck [80], and breast cancer [82], exhibit an increased expression of *MIT*/*TFE* genes, crucial transcription factors for lysosomal protein expression. These alterations in lysosomes significantly impact the proliferation and invasion of cancer cells, as well as their resilience against radiotherapy and chemotherapy [100]. The heightened activation of nutrient-scavenging pathways like autophagy and endocytosis enables cancer cells such as tumors with inadequate vascularization or those subjected to radiotherapy or chemotherapy to strive for essential nutrients and endure adverse conditions [67]. Cancer cells utilize these pathways to scavenge nutrients, activate mTOR signaling, and synthesize essential biomolecules required for unchecked proliferation [101]. Dysregulation of both catabolic and anabolic pathways creates a metabolic environment conducive to cancer progression. A feedback loop involving mTORC1 signaling and TFEB modulation orchestrates the delicate balance between lysosomal catabolism and anabolism, adapting cancer cells to varying metabolic conditions [102]. Additionally, lysosomal changes play a pivotal role in cancer cells’ evasion of immune surveillance. Lysosomal degradation not only processes antigens but also regulates the presentation of MHC-I at the cell membrane [103,104]. The lysosomal degradation of MHC-I via autophagy-dependent pathways has been implicated in the decreased surface expression of MHC-I in pancreatic ductal adenocarcinoma (PDAC). Inhibiting autophagy restores MHC-I levels, promoting T cell responses [103,104].

### 3.3. Lysosomal Calcium Regulation in Adipocytes and Ovarian Cancer Cells

The interplay between lysosomal calcium regulation and chemoresistance is an emerging area of investigation focusing on elucidating how alterations in calcium levels within lysosomes, the cellular organelles primarily responsible for waste degradation, influence the responsiveness of cancer cells to chemotherapy. Lysosomes are integral to maintaining cellular calcium homeostasis, and fluctuations in lysosomal calcium concentrations have been shown to exert regulatory effects on a multitude of cellular processes [105]. Calcium serves as a versatile signaling molecule, intricately involved in governing cell fate decisions, survival mechanisms, and responses to various forms of cellular stress [106]. Regarding autophagy-mediated cell death, lysosomes are critical for autophagy, a process that involves the degradation and recycling of cellular components [107]. Lysosomal calcium dysregulation can impact the activation of lysosomal enzymes, including those involved in drug metabolism and detoxification [108]. Moreover, alterations in lysosomal calcium levels can impact the potential to disrupt the fusion events between lysosomes and other cellular compartments, thereby influencing the intracellular trafficking of chemotherapeutic agents [109].

Our recent investigation has provided compelling evidence supporting the notion that targeting the lysosomal calcium channel TRPML1, through pharmacological or genetic means, sensitizes chemoresistant ovarian cancer cells—encompassing both established cell lines and patient-derived cells—to the effects of cisplatin treatment [19]. Specifically, lysosomal exocytosis facilitates the efflux of cisplatin from ovarian cancer cells, enabling them to evade the cytotoxic effects of the drug. Metabolomic profiling further suggests that the enhanced sensitivity to cisplatin, following inhibition of lysosomal TRPML1, is associated with a reduction in intracellular arginine levels [19]. The precise molecular mechanisms underpinning these observations warrant further investigation. Furthermore, in the context of obesity, it has been reported that TRPML1 and PPARγ are upregulated during the adipogenesis of bone-marrow-derived stromal cells [110]. These findings suggest that the process of adipogenesis may potentiate TPRML1 expression, thereby implying a potential link between obesity and the augmented function of TRPML1 in conferring chemoresistance to cancer cells. Collectively, these findings underscore the intricate interplay between lysosomal calcium dynamics and chemoresistance, offering valuable insights into potential therapeutic strategies aimed at overcoming resistance mechanisms in ovarian cancer and potentially other malignancies. Further exploration of these mechanisms holds promise for the development of novel therapeutic interventions to enhance the efficacy of chemotherapy.

### 3.4. Drug Sequestration by Dysfunctional Lysosomes in Cancer

**Figure 2:**
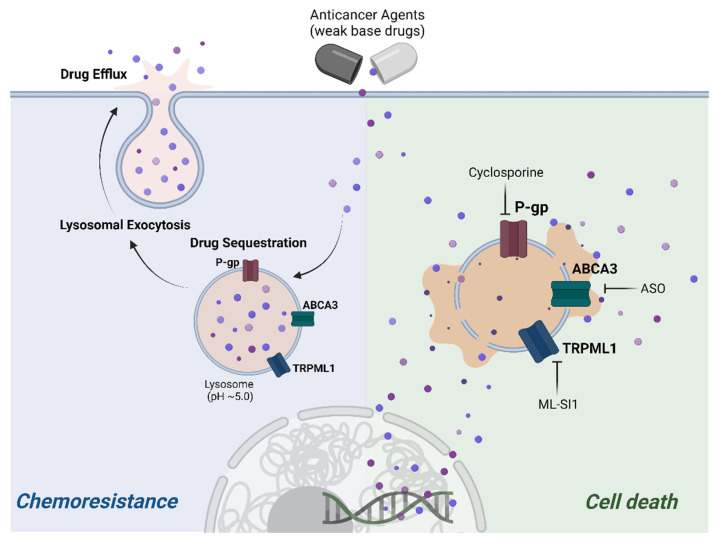
Illustration of chemoresistant mechanism of lysosomes. Pharmacological and genetic interventions targeting lysosomal channels, such as P-gp, ABCA3, and TRPML1, successfully disrupted lysosomal exocytosis in drug-resistant ovarian cancer cells, sensitizing them to treatment (P-gp, P-glycoprotein; ABCA3, ATP-binding cassette subfamily A member 3; TRPML1, transient receptor potential cation channel, mucolipin subfamily, member 1; ASO, antisense oligomer; ML-SI1, TRPML1 inhibitor). This figure was modified from a paper authored by Kim et al. in 2024 [19].

Lysosomes are integral players in the development of chemoresistance, a complex phenomenon intricately linked to the activity of P-glycoprotein (P-gp), a member of the ATP-binding cassette (ABC) transporter B subfamily responsible for the efflux of diverse substances, including drugs, from cells (Figure 2) [111]. Recent investigations have illuminated the phenomenon of P-gp overexpression within lysosomes of drug-resistant cancer cells, where it integrates into lysosomal membranes during recycling processes instead of undergoing redistribution following synthesis [112]. Cancer cells equipped with multidrug resistance (MDR) transporters efficiently remove lysosomotropic ionizing drugs, thereby sequestering them within lysosomes for subsequent release via exocytosis [113]. The accumulation of these drugs within lysosomes predominantly arises through mechanisms such as ion trapping or active transport [109]. Importantly, the comparatively weaker lysosomal membranes observed in cancer cells, in contrast to their normal counterparts, offer a potential avenue to selectively sensitize cancer cells to various forms of cell death, including apoptosis and autophagy, both of which hold considerable therapeutic significance [111].

#### 3.4.1. Sequestration of Anticancer Agents by Lysosomal Weak Bases

Anticancer drugs derived from hydrophobic weak bases, such as sunitinib, doxorubicin, daunorubicin, mitoxantrone, and imidazoacridinone, possess the capability to readily traverse hydrophobic cell and lysosomal membranes. However, upon entry into lysosomes, these agents undergo a conversion to a charged state attributed to the presence of acidic proton ions, impeding their translocation to the cytoplasm and resulting in their accumulation within lysosomes. This sequestration compromises their anticancer efficacy [114]. Moreover, the abundance of larger lysosomes commonly observed in most cancer cells enables the capture of a greater quantity of anticancer drugs, even at equivalent concentrations, thus contributing to the development of drug resistance [115]. Furthermore, lysosomal malfunction, particularly through lysosomal membrane permeabilization (LMP), can induce the efflux of sequestered anticancer agents. This phenomenon enhances sensitivity to these agents, ultimately leading to their demise.

#### 3.4.2. Anticancer Drug Sequestration via ATP-Binding Cassette Transporters

ABC transporters, abundantly distributed across the plasma membrane, play a pivotal role in the recognition and efflux of anticancer agents attempting to penetrate neoplastic cells, thereby contributing to the development of resistance against a spectrum of anticancer therapies [116]. These transporters are not solely confined to the plasma membrane but are also prominently present in lysosomal membranes, facilitating the translocation of anticancer agents from the cytoplasm into lysosomes, resulting in their sequestration and accumulation within these organelles [117]. Of particular significance is the multidrug resistance protein P-glycoprotein (P-gp), renowned for its ability to discern a wide array of chemotherapeutic agents and induce multidrug resistance in cancer cells. P-gp is not restricted to the plasma membrane but is also distributed across various intracellular organelle membranes, including lysosomes. This localization enables P-gp to contribute to the intracellular sequestration of anticancer drugs within lysosomes and other organelles, thereby conferring specific protection against these agents [113]. Additionally, ABCA3, a member of the ABC transporter family predominantly localized within lysosomes, plays a critical role in mediating anticancer drug resistance. ABCA3 facilitates the entrapment of drugs such as daunorubicin and imatinib within the lysosomal environment, thereby contributing to drug resistance mechanisms [118,119,120]. Interestingly, the absence of ABCA3 expression has been associated with heightened sensitivity to anticancer drugs, underscoring the importance of this transporter in modulating cellular responses to chemotherapy [118,121]. Moreover, recent studies have highlighted the dynamic interplay between ABC transporters and other cellular mechanisms implicated in drug resistance, including alterations in lysosomal function and intracellular trafficking pathways [121,122]. Further elucidation of these intricate interactions holds promise for the development of novel therapeutic strategies aimed at circumventing drug resistance mechanisms and improving the efficacy of anticancer therapies.

## 4. Conclusions

Our comprehensive review sheds light on the profound impact of obesity on the development of chemoresistance in ovarian cancer, focusing particularly on the lens of lysosomal dysfunction. The dysregulation of lysosomal function observed in ovarian cancer cells from obese individuals not only fosters chemoresistance but also fuels tumor growth and metastasis through various mechanisms, including impaired drug sequestration, disrupted nutrient sensing, and altered cellular stress responses. Recognizing the intricate interplay between obesity, lysosomal dysfunction, and the progression of ovarian cancer represents a crucial step towards the development of targeted therapeutic approaches aimed at overcoming treatment resistance and enhancing patient outcomes within this challenging clinical landscape. Delving deeper into the molecular intricacies underlying obesity-induced lysosomal dysfunction in cancer cells is imperative as it offers valuable insights into the mechanisms driving drug resistance. These insights pave the way for the design of innovative therapeutic interventions tailored to mitigate chemoresistance and improve treatment efficacy. Further exploration in this realm holds great promise for uncovering novel therapeutic targets and advancing the development of transformative strategies to combat drug resistance in ovarian cancer and beyond. However, this review is limited in its ability to detail precise mechanisms and introduce various research due to the nascent stage of studies exploring the association between obesity-induced lysosomal dysfunction and ovarian cancer chemoresistance.
